# Population structure, epidemiology and antibiotic resistance patterns of *Streptococcus pneumoniae* serotype 5: prior to PCV-13 vaccine introduction in Eastern Gambia

**DOI:** 10.1186/s12879-016-1370-0

**Published:** 2016-01-28

**Authors:** Eta E. Ashu, Sheikh Jarju, Michel Dione, Grant Mackenzie, Usman N. Ikumapayi, Ahmed Manjang, Romuladus Azuine, Martin Antonio

**Affiliations:** 1 Department of Biology, McMaster University, 1280 Main St. W, Hamilton, ON L8S 4 K1, Canada; 2 Medical Research Council Unit, P. O. Box 273, Fajara, The Gambia; 3 King Fahad Medical City, Central, 11525 Saudi Arabia; 4 Center for Global Health and Health Policy, Global Health and Education Projects, P. O. BOX 234, Riverdale, MD 20738 USA

**Keywords:** *S. pneumoniae*, Serotype 5, ST 3404, Population Structure, The Gambia, PCV-13

## Abstract

**Background:**

*Streptococcus pneumoniae* serotype 5 is among the most common serotypes causing invasive pneumococcal disease (IPD) in The Gambia. We anticipate that introduction of the 13-valent pneumococcal conjugate vaccine (PCV-13) into routine vaccination in The Gambia will reduce serotype 5 IPD. However, the emergence of new clones that have altered their genetic repertoire through capsular switching or genetic recombination after vaccination with PCV-13 poses a threat to this public health effort. In order to monitor for potential genetic changes post-PCV-13 vaccination, we established the baseline population structure, epidemiology, and antibiotic resistance patterns of serotype 5 before the introduction of PCV-13.

**Methods:**

Fifty-five invasive *S. pneumoniae* serotype 5 isolates were recovered from January 2009 to August 2011 in a population-based study in the Upper River Region of The Gambia. Serotyping was done by latex agglutination and confirmed by serotype-specific Polymerase Chain Reaction (PCR). Genotyping was undertaken using Multilocus Sequence Typing (MLST). Antimicrobial sensitivity was done using disc diffusion. Contingency table analyses were conducted using Pearson’s Chi^2^ and Fisher’s exact test. Clustering was performed using Bionumerics version 6.5.

**Results:**

MLST resolved *S. pneumoniae* serotype 5 isolates into 3 sequence types (ST), namely ST 289(6/55), ST 3339(19/55) and ST 3404(30/55). ST 289 was identified as the major clonal complex. ST 3339, the prevalent genotype in 2009 [84.6 % (11/13)], was replaced by ST 3404 [70.4 % (19/27)] in 2010 as the dominant ST. Interestingly, ST 3404 showed lower resistance to tetracycline and oxacillin (*P* < 0.001), an empirical surrogate to penicillin in The Gambia.

**Conclusions:**

There has been an emergence of ST 3404 in The Gambia prior to the introduction of PCV-13. Our findings provide important background data for future assessment of the impact of PCV-13 into routine immunization in developing countries, such as The Gambia.

## Background

*Streptococcus pneumoniae* is a Gram positive bacterium that colonizes the upper respiratory tract of humans and is capable of causing life-threatening infections, particularly among immunosuppressed individuals [1, 2]. *S. pneumoniae* has a circular genome containing between 2 million to 2.1 million base pairs [2]. Two-thirds of its 2236 open reading frames have been assigned roles based on their predicted gene products [2, 3]. Conserved proteins with unknown functions constitute 16 % of *S. pneumoniae*’s open reading frames, of which, 20 % are unique to *S. pneumoniae* [2, 3]. *S. pneumoniae* has 1553 and 154 genes that are important for viability and virulence, respectively. Genetic information between strains can vary by up to 10 % [2, 4].

Based on its capsular structure, *S. pneumoniae* can be categorized into more than 90 different serotypes which differ in their infection ability and severity [5, 6]. *S. pneumoniae* causes invasive pneumococcal diseases (IPD) such as septicemia and meningitis, and is the leading cause of bacterial disease in The Gambia [7]. Carriage of *S. pneumoniae* among Gambian children aged less than 1 year is 97 % and 93 % among babies aged less than 1 month [8]. Serotype 5 is one of the most common serotypes causing IPD in The Gambia [9, 10]. Pneumococcal diseases in infants and young children can be prevented by the pneumococcal conjugate vaccine (PCV). Three PCV vaccines — PCV-7 (7-valent vaccine), PCV-10 (10-valent vaccine), and PCV-13 (13-valent vaccine) — have been used in routine immunization in many countries. Even though IPD in The Gambia is commonly caused by serotype 5 [5], the 13-valent vaccine containing serotype 5 was only recently introduced in July 2011.

The incidence of IPD due to serotype 5 in Eastern Gambia in 2008 was estimated to be 7 (95 % CI 0, 38) per 100 000 person-years in children aged 2–59 months. Two years later in 2010, the incidence of serotype 5 rose to 51 (95 % CI 28, 86) per 100 000 person-years [Unpublished data, Grant Mackenzie, October 2011]. Understanding whether the introduction of PCV-13 will have an impact on the population structure, epidemiology and antibiotic resistance patterns of serotype 5 is important to scientists, public health practitioners and policy makers. The introduction of PCV-13 into the routine vaccination schedule in July 2011 is expected to lead to the reduction of serotype 5 IPD. However, the possibility of the emergence of new clones through capsular switching or recombination after vaccination is a threat to the public health impact of the vaccination program. Following introduction of PCV-7, a study in Canada showed the emergence of multi-drug resistant (MDR) serotype 19A through recombination [10]. The Canadian study painted a complex genetic picture where vaccine-selection pressure, high-level drug resistance, and *S. pneumoniae* mutational events created a “perfect storm” for the emergence of the multi-drug-resistant 19A serotype [11]. However, in order to surveil for potential post-vaccine changes, it is essential to establish a reference point from which a comparison could be made. The objective of this study was therefore to determine the population structure, epidemiology, and resistance patterns of *S. pneumoniae* serotype 5 prior to PCV-13’s introduction in to The Gambia, making available a reference point to evaluate the impact of PCV-13’s introduction into routine immunization.

## Methods

### Study area and ethical statement

The Pneumococcal Surveillance Project (PSP), hosted by the Medical Research Council Unit (MRC) in collaboration with the Gambian government is aimed at evaluating the effectiveness of the introduction of PCV into The Gambia. The PSP study area covers the south bank of the Upper River Region (1111 km^2^), which has a population of about 160,000 inhabitants [12]. The surveillance area is set in the Basse Health and Demographic Surveillance System, in which the population is enumerated every four months. The study base is located in the town of Basse. The study population comprised all cases of pneumonia, sepsis and meningitis among patients aged 2 weeks and older in the study area. This study was conducted in accordance with the Declaration of Helsinki and was approved by the Gambia Government/MRC Joint Ethics Committee (EC) — Scientific Coordinating Committee (SCC) number 1087. Written informed consent was obtained from all study participants.

### Isolation and antimicrobial susceptibility testing

Between January 2009 and August 2011, 55 serotype 5 isolates were recovered from 19 female and 24 male in a population-based study in Eastern Gambia. Isolates were recovered from either blood, cerebrospinal fluid, or lung aspirates. A total of 13 isolates were obtained in 2009, while 27 and 15 were obtained in 2010 and 2011, respectively. More than one isolate was recovered from 11 patients. Ten patients provided 2 isolates each while 1 patient provided 3 isolates. When more than one isolate was obtained from the same patient they were obtained at separate time intervals. *S. pneumoniae* was identified as previously described [13]. Oxoid antibiotic susceptibility disc (Thermofisher Scientific, UK) used to test for antimicrobial susceptibility included co-trimoxazole (25 μg), chloramphenicol (30 μg), tetracycline (30 μg), erythromycin (15 μg) and oxacillin (1 μg). Colonies of *S. pneumonie* from an overnight Mueller-Hinton agar plate supplemented with 5 % sheep blood (MHBA) were suspended in 2 ml of Mueller-Hinton broth (MHB). Tubes containing MHB were briefly vortexed to achieve a uniform suspension and turbidity was adjusted to a 0.5 McFarland standard (Biomerieux SA, France) equivalent to 1.5 × 10^8 cfu/ml. Sterile cotton tips were thereafter dipped into the suspensions and streaked to cover the entire surface of MHBA plates ensuring even spread. Antimicrobial discs were carefully placed on plates under antiseptic conditions. Plates were incubated overnight at 37 °C in 5 % CO_2_. Zones of Inhibition (ZOI) were measured and interpreted as resistant, intermediate or susceptible, using Clinical Laboratory Standard Institute (CLSI) break-point, ATCC 49619 was used as a control [13].

### Serotyping

Serotyping was carried out by latex agglutination. Briefly, for each isolate, a pneumococcal cell suspension was made in 2 ml normal saline (1.0Macfarland) from an overnight-incubated (37 °C) BA plate. Twenty microliters of each cell suspension was dispensed into 10 wells of a serotyping tray. An equivalent amount of each of the main group latex antisera (A,B,C,D,E,F,G,H,I and Omni) was added to the wells. The tray was then gently rocked for about 2 min and agglutinations observed. Serotype was determined with the aid of a chart (Statens Serum Institute, Copenhagen, Denmark). All isolates used for this project were confirmed using a serotype specific PCR. The Serotype 5 specific PCR was performed using primers targeting the wzy gene (Table 1). The portion of interest of the wzy gene was amplified as per a US Centers for Disease Control protocol [14]. Amplified PCR products were separated on 2 % agarose gel pre-stained with 0.5 μg/mL of ethidium bromide. The gel was run at 100 V in 1× Tris-borate-EDTA (TBE) for 1 h and band patterns were visualized by UV illumination with a gel documentation system (Gel Doc 2000; Bio- Rad, UK). Isolates were assigned as serotype 5 if both CpsA and serotype specific bands were amplified.

**Table 1:** Oligonucleotide primer sequences used in this study

Primers	Primer sequence (5′- 3′)	Nucleotide position^1^	Gene	GenBank accession no.
5-f	ATA CCT ACA CAA CTT CTG ATT ATG CCT TTG TG	6123^32^	wzy	CR931637
5-r	GCT CGA TAA ACA TAA TCA ATA TTT GAA AAA GTA TG	6450^32^		
cpsA-f	GCA GTA CAG CAG TTT GTT GGA CTG ACC	1473^32^	wzg	CR931662
cpsA-r	GAA TAT TTT CAT TAT CAG TCC CAG TC	1607^32^		

^1^Start position

[32]: Reference

### Genotyping (MLST)

Genotyping was done by means of Multilocus Sequencing Typing (MLST). Seven housekeeping genes including *aroE* (shikimate dehydrogenase), *ddl* (D-alanine-D-alanine ligase), *gdh* (glucose-6-phosphate dehydrogenase), *gki* (glucose kinase), *recP* (transketolase), *spi* (signal peptidase I) and *xpt* (xanthine phosphoribosyltransferase) were targeted for amplification by PCR. The PCRs were performed in 25 μl volumes per reaction as previously described [15]. Thermal cycling was performed in a Palm cycler (Corbett research) under the following conditions: 95 °C for 10 min followed by 30 amplification cycles of 95 °C for 30 s, 55 °C for 30 s, 72 °C for 1 min, and a final extension at 72 °C for 10 min. Sequencing was done by incorporation of chain-terminating dideoxynucleotides [16]. A sequencing reaction was performed for both forward and reverse strands of each targeted gene (Big Dye Terminator Cycle Sequencing kit; Applied Biosystems, UK). PCR products were purified using Qiagen purfication kit (Qiagen Sample & Assay Technologies, UK) and submitted to the in-house core sequencing facility in Fajara (Medical Research Council Unit, The Gambia).

### Data analysis

Editing and alignment of sequences for submission to MLST database [17] for allele and sequence type numbers was performed using Laser Gene DNA star, version 7.1. Data was exported into an Excel sheet (Microsoft 2007) for analyses of temporal trends. STATA version 9 (Stata Corporation, College Station, TX, USA) was used to produce summary data. The Wilcoxon signed ranked test was used to compare differences in the number of serotype 5 cases between male and female. In order to illustrate the differences between various groups with categorical data, contingency table analyses were conducted using Pearson’s Chi^2^ and Fisher’s exact test (two-sided). Genotype clustering was performed using Bionumerics version 6.5 (Applied Maths, Saint-Martens-Latem, Belgium).

## Results and discussion

### Molecular characterisation

**Fig. 1:**
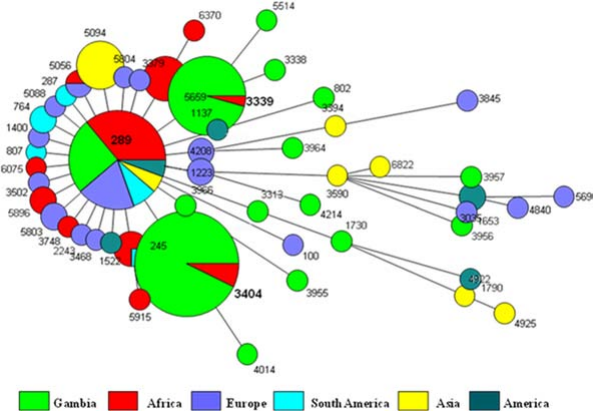
Clustering of STs from diverse world regions by use of the minimum spanning algorithm. Each circle represents an ST. The area of each circle corresponds to the number of isolates of a given ST in the MLST database. A clonal complex is a group of STs sharing a minimum of 6 out of 7 alleles [31]. The inferred putative ancestor of all clonal complexes is ST 289. Green portions represent STs found in Gambia including those used for the study while the other colours represent STs found in the rest of the world

**Fig. 2:**
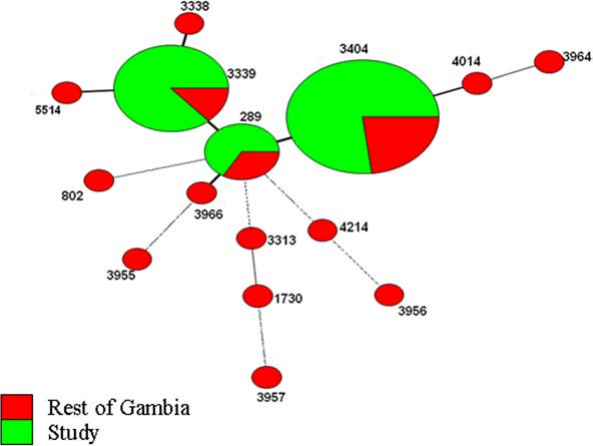
Clustering of STs from The Gambia by use of the minimum spanning algorithm. Thick, short, solid lines connect single-locus variants; thick, longer, solid lines connect double-loci variants; Thick, very long, solid lines connect three-loci variants; dash and dotted lines connect four and five-loci variants respectively. Green portions represent STs identified during the study and red portions represent ST previously described from the rest of Gambia

MLST resolved all 55 isolates into 3 sequence types (ST). ST 3404 [54.5 % (30/55)] was the most prevalent clone, followed by ST 3339 [34.5 % (19/55)] and ST 289 [11.0 % (6/55)]. In all cases where more than one isolate was obtained from the same patient, for example from blood and lung aspirate, no difference in ST was observed, suggesting that the existence of multiple STs within sterile body fluids is infrequent or non-existent. Cluster analysis on all serotype 5 isolates in the MLST database showed that both ST 3404 and ST 3339 were unique to Gambia and Senegal (Fig. 1), implying a recent and local differentiation event. ST 289 was identified as the major clonal complex in The Gambia (Fig. 2), this genotype was also widely distributed worldwide (Fig. 1). However, caution must be exercised when using circle sizes to determine the prevalence of a clone (ST), considering that submission of every IPD allelic profile to the MLST database is not obligatory.

STs 3339 and 3404 differed from ST289 at two loci, *ddl* and *spi*, respectively. *ddl* codes for an enzyme involved in cell wall biosynthesis while *spi* has been associated with the processing of signal peptides for classical MHC class I molecules [18, 19]. It would be luring to infer that the changes in *ddl* and *spi* giving rise to clones ST3339 and ST3404 are of little significance because they are non-capsular (serotype) changes. For example, *ddl* gene diversity has been linked to the recombinational replacement of penicillin binding protein 2b (*pbp2b*), a gene located 783 bp upstream of *ddl* [20]*.* This linkage suggests that changes in beta-lactam susceptibility patterns involving *pbp2b* could be reflected in *ddl* diversity. Consequently, observed disparities in *ddl* and even *spi* incidence could potentially be very important. These findings taken together emphasize the need for whole genome analysis in order to determine the relevance of the emergence of ST 3404 and the prevalence of ST3339 in The Gambia, especially because both clones originate from a recent and local differentiation event.

### Epidemiology

The median age of patients with invasive disease caused by serotype 5 was 29 months (lower quartile = 0.8 months, upper quartile = 240 months). We report no significant difference between the prevalence of ST3404 or serotype 5 — including all three clones — in male and female patients. These findings suggest that sex is not a risk factor as pertains to the acquisition of ST 3404 and of *S. pneumoniae* serotype 5 as a whole. However, these findings are not unique to serotype 5; the same has been reported about serotype 1 and most other pneumococcal serotypes that cause IPD in The Gambia [5].

**Fig. 3:**
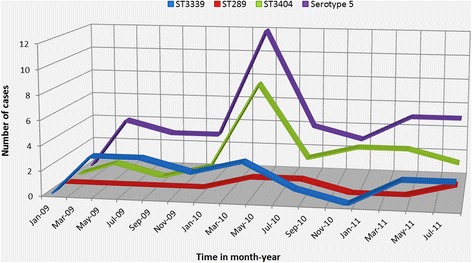
Plot showing temporal trends of serotype 5 and its observed ST’s from 2009–2011 in The Gambia. The x axis represents time in month-year with the zero point being Jan-09, whilst the y axis represents the number of serotype 5 IPDs recorded. ST 3339 was the prevalent genotype in 2009; ST 3404 peaked at the beginning of 2010 and has since been the dominant genotype for most observed IPD cases of serotype 5 origin

The number of serotype 5 cases obtained from patients with IPD varied from year to year, with the most number of cases reported in between February and June 2010. The increase in number of serotype 5 in 2010 was correlated with a slight decrease in the prevalence of ST 3339 and the emergence of ST3404 (Fig. 3). ST 3404 peaked at the beginning of 2010 and has since then been the prevalent ST responsible for most serotype 5-caused IPD. A number of observations could be advanced on why ST 3404 has emerged. For example, it’s plausible to hypothesize that the introduction of PCV-7 in August 2009 might have indirectly acted as a selective pressure for the emergence of the new clone (ST 3404). However, the unavailability of detailed epidemiological information which could potentially explain why there is an increase in incidence of ST 3404 in this study calls for caution in accounting for the observed trend. Further research is needed to explain the observed trend.

The most number of serotype 5 isolates collected were recorded between the months of May to June each year. These seasonal peaks could be explained by the fact that in The Gambia this period marks the end of the dry season. During the dry seasons in the West African belt, dry and dusty winds damage the mucosa membrane of the oral cavity [21]. Low absolute humidity and dust inhibit the mucosal immune defenses by damaging the mucosal membrane leading to an increased risk of transmission during the dry season. Transmission capacities of other bacterial pathogens such as Meningococcus have been reported to be less during the rainy seasons due to higher humidity [22, 23]. Serotype 5, like most other serotypes in The Gambia, peaks after the dry and hot season [15]. Given that Meningococcus and Streptococcus are both transmitted by close contact with respiratory fluids, assessment of the contribution of low absolute humidity and dust as risk factors for outbreaks of *S. pneumoniae* is of interest in The Gambia.

### Antibiotic resistance

All 55 *S. pneumoniae* serotype 5 isolates were resistant to co-trimazole. Among these isolates, 44 % (24/55) and 73 % (40/55) were susceptible to tetracycline and oxacillin, respectively. Furthermore, 98 % (54/55) of all isolates were susceptible to chloramphenicol. Observed chloramphenicol susceptibility is consistent with a previous study in The Gambia which showed that all invasive *S. Pneumoniae* serotype 1 isolates were susceptible to chloramphenicol. We however note that susceptibility patterns vary slightly by serotype with respect to tetracycline, co-trimazole, and oxacillin [15]. The proportion of serotype 5 isolates susceptible to tetracycline between January 2009 to April 2010 was 0.34 (10/29) while that between May 2010 to August 2011 was 0.62 (16/26). Compared to the period between January 2009 to April 2010, we found that the number of isolates susceptible to tetracycline significantly increased between May 2010 to August 2011 (Pearson chi^2^ = 0.02, Fisher’s exact test = 0.03). The same was true for oxacillin although the increase in susceptibility was not statistically significant. The proportion of ST 3404 isolates susceptible to tetracycline and oxacillin was 0.83 (25/30) and 0.97 (29/30), while that for ST 3339 was 0.05 (1/19) and 0.37(7/19), respectively. There is strong evidence that the number of isolates susceptible to tetracycline and oxacillin is different for ST 3404 and ST 3339, with the emerging clone ST3404 showing greater susceptibility. All P-values for both Pearson chi^2^ and Fisher’s exact test were < 0.001.

Resistance to 3 or more classes of antibiotics is commonly referred to as multiple-antibiotic resistance (MDR) [24]. About Sixty-one percent (8/13) of all isolates collected in 2009 were resistant to multiple antibiotics, while 30 % (8/27) of all isolates in 2010 and 7 % (1/15) in 2011 were resistant to multiple antibiotics, implying a decrease in the prevalence of MDR serotype 5 over time. Overall, only one ST 3404 isolate showed MDR, suggesting the drop in MDR over time could be attributed to the emergence of ST 3404. Studies carried out in Canada, Greece, Sweden and Europe as a whole reveal that antibiotic resistance patterns are dependent on local prescribing patterns [25–28]. Drug administration patterns such as dose, route of administration, and duration of antibiotic regimen also influence antibiotic resistance [26]. However, the molecular basis of antibiotic resistance is a major factor to consider since it influences the likelihood of selection. Resistance requiring the introduction of a gene from a donor species is less likely to occur than resistance mediated by a single DNA base change, e.g. pneumococcal resistance to rifampin and fluoroquinolones [26]. Results from our study are similar to those from a study in Canada where decline in MDR 19 F, 6B, and 23 F were documented post PCV-7 [11]. The above study suggested that among other factors, vaccine pressure could influence changes in antibiotic susceptibility patterns.

The emergent strain in this study, ST3404, allegedly showed increased susceptibility to tetracycline, oxacillin, and consequently penicillin. In The Gambia, oxacillin is used as an empirical surrogate to penicillin; likewise it is predictive of reduced penicillin susceptibility [29]. However, caution must be exercised when using oxacillin disc diffusion to predict penicillin susceptibility patterns as predictions can at times be limited by false susceptible and resistance interpretations [29]. Nonetheless, by reason of the magnitude of the difference in susceptibility to oxacillin shown by STs 3404 and 3339 (*P* < 0.001) and the overall low interpretive error rate of oxacillin disc diffusion [29, 30], ST 3404’s alleged increased susceptibility to penicillin might be genuine. In light of these results, surveillance to determine if these patterns will change after the introduction of PCV-13 will be vital to highlight the role of immunization on susceptibility patterns of various serotype 5 clones (ST) in The Gambia.

## Conclusion, implications and future perspectives

There was an emergence of ST 3404 in The Gambia prior to the introduction of the PCV-13. Interestingly, compared to ST 3339, ST 3404 showed lower resistance to tetracycline and oxacillin. Findings from this study provide important background data to assess the potential impact of PCV-13 in routine immunization in The Gambia. Finally, we recommend a comparative genome study on STs 3404, 3339 and 289 to better understand differences antimicrobial sensitivity and virulence, if there be any.
